# Bioformulation of Silk-Based Coating to Preserve and Deliver *Rhizobium tropici* to *Phaseolus vulgaris* Under Saline Environments

**DOI:** 10.3389/fpls.2021.700273

**Published:** 2021-08-02

**Authors:** Manal Mhada, Augustine T. Zvinavashe, Zakaria Hazzoumi, Youssef Zeroual, Benedetto Marelli, Lamfeddal Kouisni

**Affiliations:** ^1^African Integrated Plant and Soil Research Group, AgroBioSciences, Mohammed VI Polytechnic University (UM6P), Benguerir, Morocco; ^2^Department of Civil and Environmental Engineering, Massachusetts Institute of Technology, Cambridge, MA, United States; ^3^Green Biotechnology Laboratory, Moroccan Foundation for Advanced Science Innovation and Research (MAScIR), Rabat, Morocco; ^4^Situation Innovation—OCP Group, Casablanca, Morocco; ^5^African Sustainable Agriculture Research Institute (ASARI–UM6P), Laayoune, Morocco

**Keywords:** biomaterial in agriculture, sustainable agriculture, food security, soil salinity, seed coating

## Abstract

Seed priming has been for a long time an efficient application method of biofertilizers and biocontrol agents. Due to the quick degradation of the priming agents, this technique has been limited to specific immediate uses. With the increase of awareness of the importance of sustainable use of biofertilizers, seed coating has presented a competitive advantage regarding its ability to adhere easily to the seed, preserve the inoculant, and decompose in the soil. This study compared primed *Phaseolus vulgaris* seeds with *Rhizobium tropici* and trehalose with coated seeds using a silk solution mixed with *R. tropici* and trehalose. We represented the effect of priming and seed coating on seed germination and the development of seedlings by evaluating physiological and morphological parameters under different salinity levels (0, 20, 50, and 75 mM). Results showed that germination and morphological parameters have been significantly enhanced by applying *R. tropici* and trehalose. Seedlings of coated seeds show higher root density than the freshly primed seeds and the control. The physiological response has been evaluated through the stomatal conductance, the chlorophyll content, and the total phenolic compounds. The stability of these physiological traits indicated the role of trehalose in the protection of the photosystems of the plant under low and medium salinity levels. *R. tropici* and trehalose helped the plant mitigate the negative impact of salt stress on all traits. These findings represent an essential contribution to our understanding of stress responses in coated and primed seeds. This knowledge is essential to the design of coating materials optimized for stressed environments. However, further progress in this area of research must anticipate the development of coatings adapted to different stresses using micro and macro elements, bacteria, and fungi with a significant focus on biopolymers for sustainable agriculture and soil microbiome preservation.

## Introduction

Seed production is one of the critical agri-businesses that relies not only on exploiting potential genetic resources of the crop-seed itself but also on advances in multiple technologies that enable efficient use of different biological resources such as beneficial microorganisms.

Seed priming or pretreatment was used in agriculture a century ago. However, the use of bioagents was first reported by Callan et al. (1990). The method implies seed inoculation with beneficial microorganisms. According to Bisen et al. (2015), the biopriming with beneficial microbes offers an innovative crop protection tool by improving the seed quality, germination rate, seedling vigor and uniformity, and plant ability to withstand harsh growth conditions, thus ensuring sustainable crop production (Bisen et al., 2015). Another use of biopriming has sprung by understanding plant growth promoting bacteria functions and mechanisms. The discovery of phosphate solubilizing bacteria has led to its use in agriculture as a potential biofertilizer. For example, it has been reported that phosphate solubilizing rhizobacteria strongly influences wheat root traits and aboveground physiology (Elhaissoufi et al., 2020). Although seed priming is facing many challenges related to the conservation of bioformulation, storage, and difficulties in upscaling management. We can overcome challenges by coating seeds using biofertilizers embodied within films synthesized from biomaterials (Zvinavashe et al., 2021).

Among the most important features that a germinating seed may exhibit would be a coating agent that preserves seed health, guarantees a high germination rate, tolerates stresses, delivers beneficial microorganisms, and contributes to sustainable agriculture (Pedrini et al., 2017; Rocha et al., 2019). Thus, the importance of biopolymers designed for agriculture and inspired by nature. Silk was one of the most promising biopolymers due to its natural properties. The combination of beta-sheet structures with inter-and intra-molecular hydrogen bonds that provides high flexibility in the natural fiber also guarantees extreme conformability in regenerated film format (Marelli et al., 2016).

Many research activities are conducted to develop a good coating agent and components that could play a role in crucial stages in plant growth in stressed environments (Maity et al., 2019; Soumare et al., 2020; Meftah Kadmiri et al., 2021). To develop new strategies to enhance abiotic stress tolerance of crop plants (Iordachescu and Imai, 2008), bioinspired coating using silk was developed (Zvinavashe et al., 2019), combining trehalose and *R. tropici*. Both have been known for their effect on different stages of the crop cycle and their ability to promote growth.

Trehalose has been identified as an osmoprotectant and signaling molecule controlling various processes ranging from seed development and germination to guard cell movement and overall plant growth (Gómez et al., 2010). Trehalose is biosynthesized through different pathways in different living organisms except for mammals (Elbein et al., 2003). The catabolism enzymes are trehalase, trehalose phosphorylase, trehalose-6-phosphate phosphorylase, and trehalose-6-phosphate hydrolase which are widespread in living cells and may be of considerable importance in regulating the level of trehalose in the cell and managing the trehalose/sugar ratio in the demanding sink (Chen and Haddad, 2004). The level of trehalose is significantly increased in various cells when exposed to various environmental stresses, reflecting that abiotic stresses differentially regulate trehalose biosynthesis genes in higher plants (Bae et al., 2005).

The second component of the newly designed coating called “Silk Coating” is *R. tropici*. Besides its ability to promote growth and increase stress tolerance in legumes, trehalose accumulation has also been described in *Rhizobium* in response to several stressful conditions such as high external osmolarity, low oxygen concentrations, or desiccation (McIntyre et al., 2007). Trehalose also acts as an osmoprotectant when exogenously supplied to *R. leguminosarum* (Gouffi et al., 1999). It is also a common disaccharide in the root nodules of legumes and is present in bacteroids serving as molecular signals during biological nitrogen fixation (Gómez et al., 2010; Zvinavashe et al., 2019), which could reflect an important role for this molecule in symbiosis. It was also demonstrated by Müller et al. (1998) that the addition of exogenous trehalose to the growth medium increased sucrose synthase and alkaline invertase activities in soybean. Besides, the inoculation of common bean (*Phaseolus vulgaris*) with the symbiotic bacterium *R. etli*, which was engineered to overexpress the *Escherichia coli* trehalose-6-phosphate synthase (otsA), resulted in more trehalose production and formation of more nodules with higher nitrogenase activity (Suárez et al., 2008). In agreement with this, deletion of the endogenous otsA gene in *R. etli* reduced the number of nodules, nitrogenase activity, and plant biomass.

Due to the well-known stress-protecting properties of trehalose in microorganisms and plants, “trehalose/bacteria” could be a valuable mixture to target to improve abiotic stress tolerance in crop plants.

The objectives of this study were (1) to investigate the effect of exogenous application of trehalose and *R. tropici* in two different methods (Priming and coating) on different parameters; (2) to compare the freshly primed *P. vulgaris* seeds with trehalose and *R. tropici* with seeds coated with trehalose and *R. tropici* using silk as a coating agent; and (3) and to explore the potential of silk as a coating agent.

## Materials and Methods

### Plant Material

Seeds of common bean (*P. vulgaris*) commercial variety White Kidney, with an initial germination rate above 95% and initial seed moisture content below 10% (on a dry weight basis), were used in this study. To minimize fungal contamination during the experiment, seeds were surface sterilized with 2% NaOCl solution (household bleach diluted with sterile water) for 15 min and rinsed three times with sterile water.

**Figure 1:**
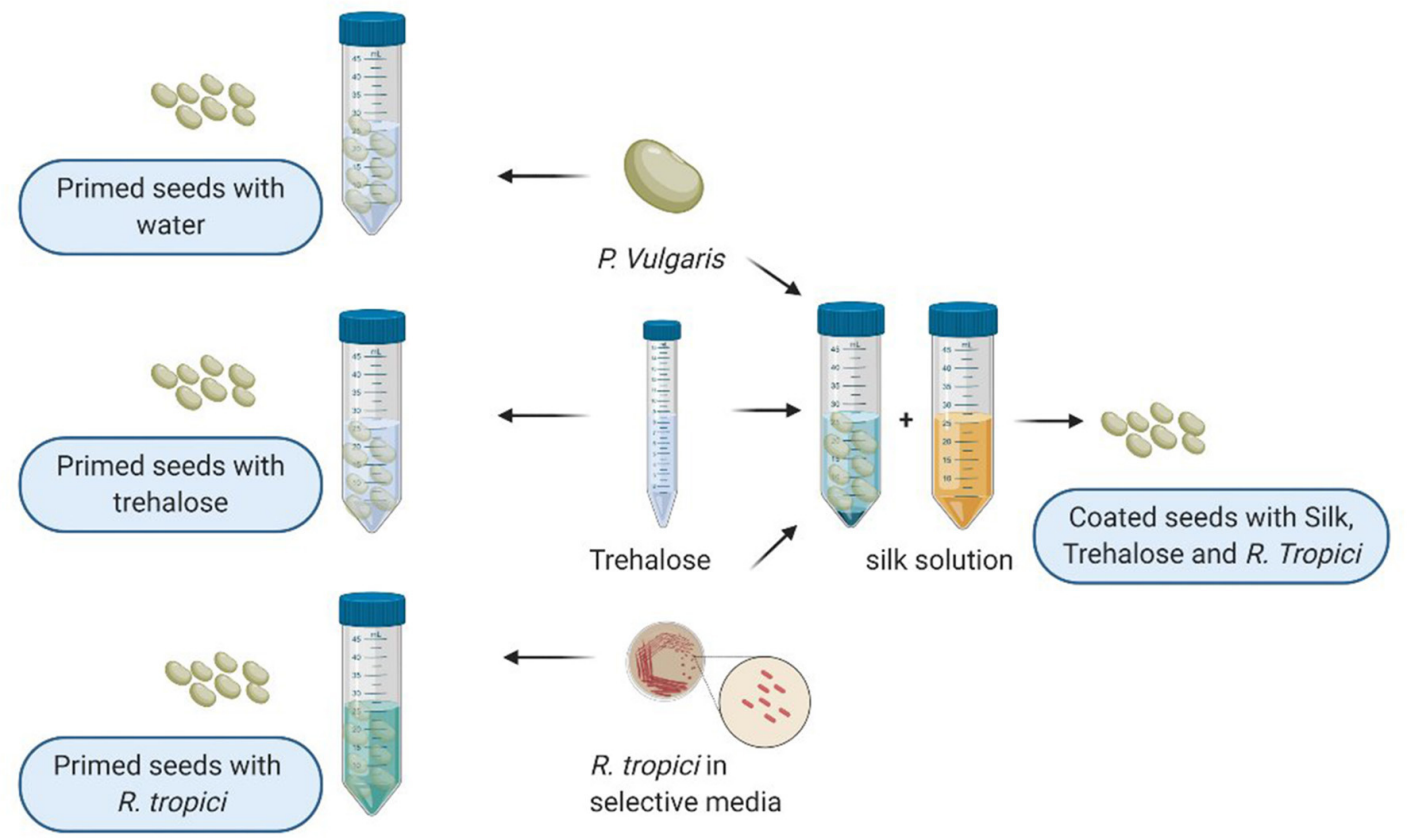
Illustration of seed priming and seed coating protocol created with BioRender.com.

Before seed preparation, the coating solution was prepared using the protocol developed by Zvinavashe et al. A 6% silk solution was prepared based on Rockwood et al. (2011) protocol to which we added trehalose at 6% and *R. tropici* grown in Lysogeny broth (LB) media at 28°C until achieving an OD_600_ value of 2. The bacterial solution was centrifuged for 5 min. The pellet was rinsed two times using sterile water, as shown in Figure 1. Before usage, seeds were stored at cool temperature (4°C) for 70 days.

### Experimental Details

In addition to seeds coated with silk, trehalose, and *R. tropici* (named: Coating) previously described, healthy seeds were primed at the laboratory using sterile water for the negative control and Trehalose 6% and *R. tropici* (cultivated at LB media and watched and centrifuged twice with distilled and sterilized water) as a positive control with constant gentle agitation for 30 min (Figure 1). To ensure that the priming solution covered all the seed surfaces, a volume of 1:5 is used for negative and positive control treatments. This methodology was inspired by *On-farm* seed priming described by Harris et al. (2001).

To assess the effect of each treatment on common bean germination, 15 healthy seeds from each treatment were evenly germinated on two layers of filter paper in 10 cm diameter Petri dishes. After adding 10 mL of NaCl solutions (*S0* = 0, *S1* = 15, *S2* = 25, and *S3* = 50 mM) to each replicate, Petri dishes were covered with lids and placed in the dark. An equal volume of distilled water was applied to all Petri dishes when their moisture content declined. All the treatments were laid out in a randomized complete block design, replicated three times, and kept recording physiological attributes.

To ascertain the role of seed priming in alleviating salinity, the pots experiment was conducted using large pots (20/30 cm) filled with local soil without any physical treatment. The four previous seed treatments were applied to determine the effect of salinity on the plant establishment and behavior along the growth cycle. Five salinity levels (0, 25, 50, 75, and 100 mM) were applied.

### Morphological and Physiological Response of the Seed and Plant

During the experiment, different levels of analysis were conducted to dissociate the components of the combined effects of the treatment. At the seed level: (1) daily germination and (2) germination rate. At the shoot level: (1) shoot length; (2) stomatal conductance using an SC-1 Leaf Porometer (Decagon Devices, Inc., Pullman, WA); and (3) chlorophyll content using the CL-01 Chlorophyll Content System. Moreover, at the root level, (1) root density; (2) primary root length; and (3) lateral root length were measured using the root scanner WinRhizo (Regent Instruments Inc., Quebec, Canada).

### Biochemical Response

#### Extraction of Total Phenolic Compounds (TPC)

Fragments of leaves and roots (0.5 g FM) were ground in a mortar containing a specific volume, usually 5 mL, of ethanol 50% (water-alcohol solution). The extracts were collected in tubes with lids and well-numbered, then left overnight at 4°C to let ethanol extracting the maximum amount of phenol present in the leaves extract as described by Singleton and Rossi (1965). In the tubes containing the leaf extracts, there was a risk of the existence of chlorophylls, eliminated by adding 3 mL of the extract on 0.5 mL of chloroforms. Tubes were vortexed and centrifuged for 5 min at 5 × 1,000 mtp; two phases were separated, supernatant was added, and pellet.

#### Determination of TPC

We followed the method based on the Folin–Ciocalteu reagent, described by Singleton and Rossi (1965) and Hazzoumi et al. (2017). The following mixture was prepared in test tubes: 0.5 mL of extract, 3 mL of distilled water, 0.5 mL of Na2CO3 (20%), to which 0.5 mL of Folin–Ciocalteu reagent was added after 3 min. The tubes were agitated and then placed for 30 min at 40°C. After that, the absorbance was read at 760 nm. The content of phenolic compounds was calculated using gallic acid for the standard curve and expressed in milligrams per gram of fresh leaf matter.

### Statistical Analysis

A two-way ANOVA was performed using the general linear model to compare seed treatments and saline levels. Descriptive statistics and ANOVA were carried out using the software R version 3.6.0 with the Agricolae, followed by Tukey's multiple comparison test (*p* < 0.05) to discriminate statistically different values. Graphs were generated using the ggplot2 in R version 4.0.5 version.

## Results

### Germination and Seedlings Development

Seed germination is a critical phase that impacts crop establishment and yield. It is considered a bottleneck, and once surpassed, the plant can use other strategies to tolerate an excess of salt in the environment. To evaluate the effect of salinity on seed germination and seedlings, a daily record was conducted. ANOVA test (Table 1) shows that seed treatment and salinity significantly affect germination throughout the experiment. A highly significant interaction was observed between the treatments.

**Table 1:** ANOVA table of the evolution of germination throughout the experience, germination rate (GR), and hypocotyl diameter (HD) in early germination test.

	**df**	**Day 2**	**Day 3**	**Day 4**	**Day 5**	**GR**	**HD**
Seed Treatment	5	0.090^.^	0.046^*^	0.0436^*^	0.130	0.0240^*^	5.41e-08^***^
Salinity level	3	0.009^**^	0.020^*^	0.0128^*^	0.001^**^	0.0018^**^	7.84e-07^***^
Seed Trt: Salinity	15	0.174	0.002^**^	0.0009^***^	0.003^**^	0.0002^***^	0.0265^*^
Residual	48						

*Signif. codes: 0*

‘***’ *0.001*

‘**’ *0.01*

‘*’ *0.05*

‘.’ *0.1 ‘’ 1*.

Table 2 illustrates the effect of increasing salt (0 mM to 50 mM) on the percent of germinating seeds. It was observed that the percentage of germination decreases with increasing salt concentrations for the control seeds. However, a significant increase happens when applying a seed treatment. The ANOVA (Table 1) showed that coated seeds behave better at a high salinity level than the freshly treated seeds because the silk preserves the bacteria and the trehalose in its fibroins.

**Table 2:** Means and SD of *Phaseolus vulgaris* germination rate (GR) in percent under different seed treatments and salinity levels.

**Salinity levels**	**Control**	***R. tropici***	**Trehalose**	**Coating**	**Means**
S0	87 ± 9^a^	87 ± 9^abc^	80 ± 16^cd^	87 ± 9^abc^	85 ± 12
S1	87 ± 9^abc^	80 ± 0^cd^	93 ± 9^ab^	87 ± 9^abc^	87 ± 9
S2	67 ± 9^cd^	87 ± 9^abc^	87 ± 9^abc^	93 ± 9^ab^	83 ± 14
S3	53 ± 9^d^	73 ± 9^cd^	87 ± 9^abc^	93 ± 9^a^	77 ± 18
Means	73 ± 17	82 ± 10	87 ± 12	90 ± 10	83 ± 14

a–d *Different letters indicate significant statistical differences (p < 0.05, Tukey's test)*.

**Figure 2:**
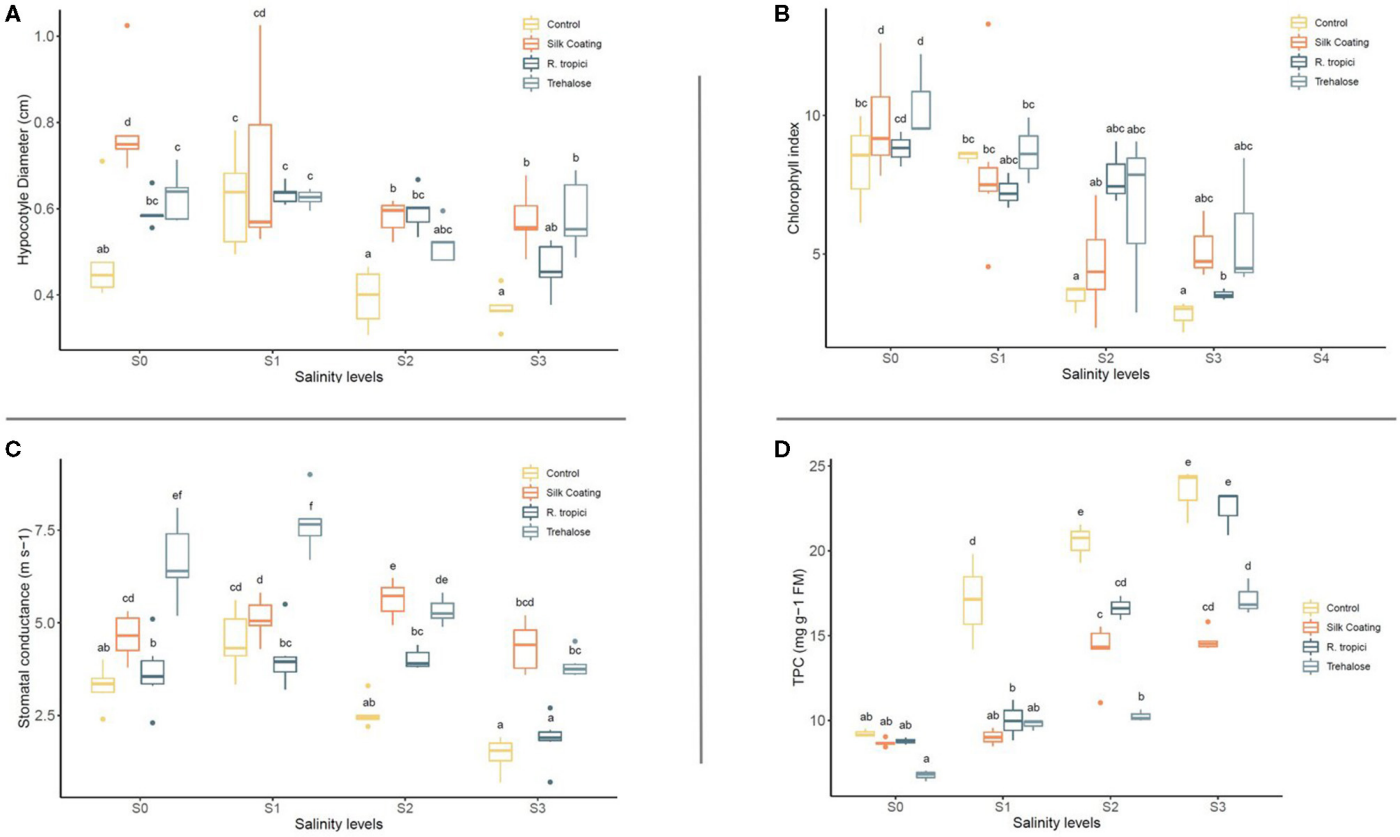
Effect of seed treatments on hypocotyl diameter **(A)**, stomatal conductance **(B)**, chlorophyll content **(C)**, and total phenolic compounds [TPC, **(D)**], under different salinity levels (*S0* = 0 mM, *S1* = 25 mM, *S2* = 50 mM, and *S3* = 75 mM).

Besides germination rate, ANOVA analysis showed that hypocotyl diameter (HD) varies significantly between seed treatments and saline levels (Table 1). Presented data showed that salinity stress distinctly decreases hypocotyl diameter (Figure 2). However, the seed treatment affects the behavior of the seedling and induces an increase in hypocotyl diameter. At a low salinity level (S1), the Silk Coating presents the largest diameter with 0.67 cm, followed by trehalose and bacteria (0.50 and 0.46 cm, respectively). The same trend is observed at 15, 25, and 50 mM of salinity levels.

At S2 (25 mM), the common bean is intensely influenced by salinity, and seed treatments reinforce the seedling by increasing the hypocotyl diameter. At S3, the highest salinity level (50 mM), the inoculated seed with *R. Tropici*, presented a diameter equivalent to the control seeds. The Silk Coating and trehalose remained higher with 0.45 and 0.44 cm. Nevertheless, the control presents the lowest diameter in all salinity levels. At 15 mM, salinity concentration stimulates the growth for the control, indicating that salt at low concentration can be used as a priming agent even for glycophytes.

### Physiological Activity

The ANOVA for different physiological parameters showed that the effects of seed treatments and salinity on common bean plants are significant (Table 3).

**Table 3:** ANOVA table of physiological parameters measured from pot experiment.

	**df**	**SEm**	**StC**	**Chll**	**TPC**
Seed Trt	3	0.0102^*^	1.64e-14^***^	0.0001^***^	0.0073^**^
Salinity level	3	3.54e-16^***^	1.84e-12^***^	<2e-16^***^	4.53e-06^***^
Seed Trt:Salinity	9	0.0301^*^	3.14e-07^***^	0.0003^***^	0.1214
Residual	80				

*Signif. codes: 0*

‘***’ *0.001*

‘**’ *0.01*

‘*’ *0.05*

*‘.’ 0.1 ‘’ 1*.

*SEm, seed emergence; Chll, chlorophyll content; StC, stomatal conductance; TPC, total phenolic compounds*.

#### Stomatal Conductance

The effects of salinity on stomatal conductance are shown in Figure 2. Stomatal conductance always declines with increasing salinity concentration. The effect of salinity was more dramatic in control seeds than in the other treatments. At S3 (50 mM), Silk Coating performs better than seeds treated with trehalose and the bacteria, while the control remains at the last position, indicating that plant reaction to salinity relies on the seed treatments.

#### Chlorophyll Content

Data for stomatal conductance as a gas exchange characteristic and a level of plant control showed that, upon exposure to saline stress, the plant significantly reduces its photosynthetic activity through the control of the stomata. Damages caused by salinity are felt at the photosynthesis machinery. Data presented in Figure 2 shows that the application of saline stress significantly reduced the photosynthetic activity at the branching and vegetative growth stage of the common bean. This decrease in the chlorophyll index was more intense for the control as compared with the other treatments. However, freshly treated seeds presented a high photosynthetic activity in comparison with the coated seeds.

At S0, seeds soaked in trehalose showed the highest photosynthetic activity, followed equally by the seeds soaked in the bacteria, the Silk Coating, and the control. By increasing the salinity level to 25 mM, the plant activity decreases slightly compared with 50 mM salinity level where the control reduces its photosynthetic activity to half, while the treated seeds with *R. tropici* and trehalose maintain their activity. Silk Coating and trehalose treated seeds showed a different reaction compared to the control and the bacteria in 75 mM because of the existing charge of bacteria and trehalose that could be preserved in the silk fibroins.

#### TPCs

Content of TPC changes with the coating application, trehalose, and salinity level. According to Figure 2, the quantity of phenolic compounds in the control plant increases progressively with salinity levels, reflecting a natural response of the plant against the ions of the salt in the medium. However, the seeds soaked in the trehalose reflect a significant reduction of the TPC level. The soaked seeds with bacteria presented the best response against salts in the medium. By recording the most significant decrease of TPC, the content of this latter was less than the control, which means that the plant did not need to synthesize more TPC to tolerate salt stress.

The biosynthesis of phenolic compounds is proportionally linked to salts concentration in the plant microenvironment, whatever the treatment is. About 0 mM showed one-third <75 mM; this variation keeps the same trend in *R. tropici* and Silk Coating, but the trehalose showed a different reaction for 25 mM and 50 mM salinity levels. These treatments reflect almost the same TPC content, highlighting the ability of the trehalose ability to mitigate the negative impact of salt stress on the plant up to an optimal concentration (50 mM in this case).

Using a formulation based on Silk material enriched by *R. tropici* helped the plant to tolerate salt stress, especially in the lowest and highest concentrations; however, the trehalose showed a significant effect in the medium salt stress concentration (50 mM).

### Morphology of the Plant

#### Length of the Shoot

**Figure 3:**
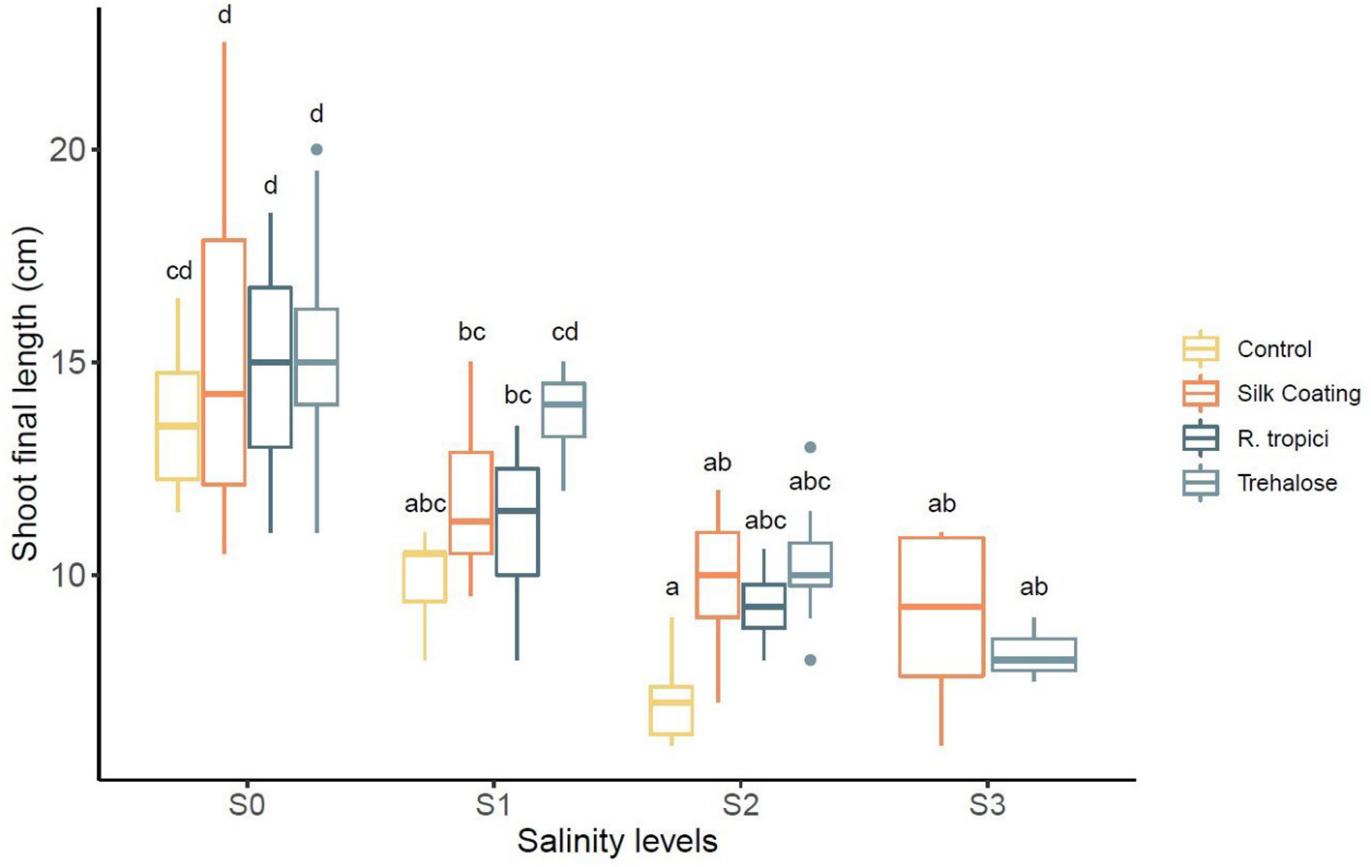
Effect of different salinity levels (*S0* = 0 mM, *S1* = 25 mM, *S2* = 50 mM, and *S3* = 75 mM) on *Phaseolus vulgaris* shoot length in centimeters.

Application of salt stress significantly reduced the plant length for all seed treatments (Table 4). A similar decreasing trend was observed due to salt stress. However, this decrease was not the same in all seed treatments. The control seeds were more severely affected by salinity than were the primed and coated seeds. The trehalose and the bacteria significantly increased the length under stress and non-stress conditions (Figure 3).

**Table 4:** ANOVA table of *Phaseolus vulgaris* morphological parameters measured from pot experiment.

	**Df**	**SFL**	**RD**	**FRL-P**	**FRL-L**
Seed Trt	3	0.00898^**^	4.81e-11^***^	2.01e-15^***^	<2e-16^***^
Salinity level	3	<2e-16^***^	7.15e-07^***^	7.76e-08^***^	<2e-16^***^
Seed Trt:Salinity	9	0.65123	0.0553^.^	0.987	2.31e-15^***^
Residual	80				

*Signif. Codes: 0*

‘***’ *0.001*

‘**’ *0.01*

‘*’ 0.05

‘.’ *0.1 ‘’ 1*.

*SFL, shoot final length; RD, roots density; FRL, root final length; P, primary; L, lateral*.

#### Architecture of the Root

##### Density of the Root

**Figure 4:**
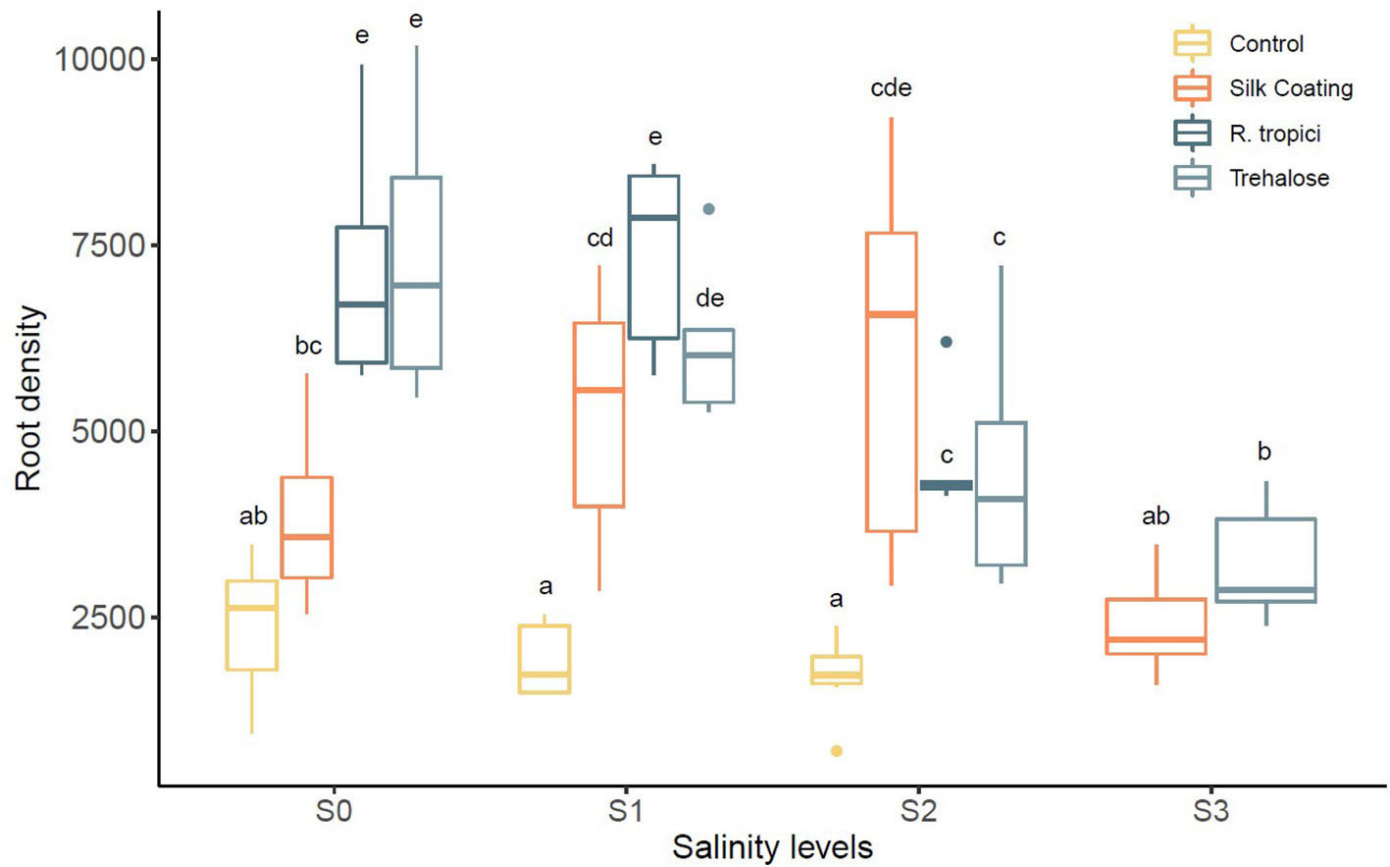
Effect of different salinity levels (*S0* = 0 mM, *S1* = 25 mM, *S2* = 50 mM, and *S3* = 75 mM) on *Phaseolus vulgaris* root density.

At different salinity levels, seed treatments have increased the root density compared to the untreated control (Figure 4). Nevertheless, data indicate that gradually increasing salinity levels from 0 to 75 mM NaCl has decreased root density. The application of trehalose induces an additive stimulatory effect on roots. The same growth pattern was observed for seeds freshly inoculated with *R. tropici*. However, the effect is reduced with increased salinity level testifying to the impact of salinity on the bacterial development in soil. In addition, it was noticed that the Silk Coating has a more pronounced and significant increase of roots than the control. At 75 mM of NaCl, the uncoated control and the seed treated with the bacteria did not tolerate the high salinity level and died after 35 days. In contrast, seeds treated with trehalose and coated seeds are behaving well and produce mature plants. This salt concentration is a threshold concentration for common bean; the cultivar used in this experiment does not survive beyond it. The plant viability at this salt concentration may be due to trehalose that promotes roots formation and growth at an early stage. The coated seeds with silk also showed viable plants, and this could be evidence of the role of the silk-based coating on the protection of the bacteria in a high salinity environment.

##### Primary and Lateral Roots

**Figure 5:**
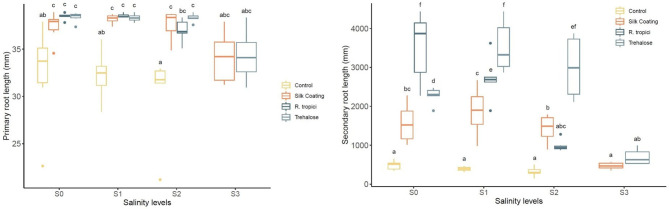
*Phaseolus vulgaris* primary roots length (left) and lateral root length (right) under different seed treatments and salinity levels.

After 90 days of growth, it was observed that common bean seedlings produce a highly branched root system with abundant lateral roots and a short primary root. Changes in root parameters of common bean under three seed treatments are presented in Figure 5. Response to seed treatments did not differ significantly for primary root length under stress and non-stress conditions. However, they are significantly higher than the control.

Irrigation of common bean plants with saline water caused significant decreases in root development relative to control plants. On the other hand, plants from treated seeds enhanced lateral root length under unstressed and salinity-stressed environments. At a low salinity level, inoculation with *R. tropici* was more effective, followed by trehalose. Coated seeds have presented the same principal root length but less lateral root length. Even though it has better performance than the control at unstressed and salinity-stressed conditions, these results suggest that, in *P. vulgaris*, Silk Coating, trehalose, and *R. tropici* may play a crucial role in changing root architecture under saline stress.

## Discussion

### Germination and Seedlings Development

Germination is a critical stage in the crop cycle. It has been demonstrated that this stage is susceptible to all stresses. In this study, the effect on germination rate induced by seed treatment underlines the importance of priming and biopriming in seed production to improve plant behavior under stressed environments. To alleviate saline stress, a seed coating was developed using Silk as a binding agent, trehalose for its ability to stimulate germination and growth in saline stress and *R. tropici* for its ability to colonize common bean roots and produce nodules for an efficient Nitrogen fixation (Zvinavashe et al., 2019).

Seed priming is one of the universal methods used in fertilizers application. Freshly primed seeds usually contain a high concentration of the priming agents, but it is susceptible to degradation or leaching after irrigation. Results are proof of the efficiency of the Silk Coating since it exceeds all the seed treatments, mainly in the high salinity levels by maintaining the trehalose and the bacteria in the seed microenvironment. It has also shown a high germination rate and high seedlings diameter at the highest salinity level, supporting the hypothesis of the conservation of the formulation elements by the Silk fibroins in saline conditions.

To further understand the priming effect on germination, independent experimentation was conducted *in vitro* using a Petri dish to evaluate the germination rate and the seedling vigor under saline treatments. The same trend was observed, and the coated seeds performed better than the other treatments for inducing germination in saline treatments. Besides, it confers to the seedling a significant vigor, explaining the plant strength during the growth cycle. The observed effects are mainly due to the combined action of trehalose and *R. Tropici*, both of which promote growth through different mechanisms. Trehalose, a disaccharide, is an essential signal metabolite in plants and an essential regulator of many physiological processes, including seed germination and stress response. Within the seed germination process, trehalose plays an essential role in regulating the growth of vegetative and embryogenic tissue in a mechanism involving ABA and sugar metabolism (Gómez et al., 2010). It also plays a vital role as an osmolyte, stress signaling, and stress protectant of the membrane and proteins against the adverse effects of stress (Crowe et al., 1984).

Trehalose regulates many genes, among them the transcription factor bZIP11, which affects the regulation of carbohydrate metabolism, inducing a regulation of the developmental phase transitions (like germination and flowering), carbohydrate, and amino acid metabolisms (Gazzarrini and Tsai, 2014). Accordingly, it has been reported that, in rice, the [trehalose:sucrose] ratio in germinating tissues leads to a regulation of amylase activation for increased starch mobilization and contributes through its regulators OsTPP7 to anaerobic germination tolerance (Kretzschmar et al., 2015). Trehalose hydrolysis is a major reaction during early germination. Presumably, it serves as a source of carbon for synthesis and glucose for energy, and its use in seed coating has been efficient.

*R. tropici* was added to the silk and trehalose to increase the efficiency of the coating formulation. This bacterium has been widely studied and has proven its ability to increase yield components during the crop cycle, even in areas with medium salinity where untreated seeds do not perform very well. However, it has been observed that it is sensitive to high salinity. Therefore, an exogenous application of trehalose may also help the bacteria tolerate abiotic stress (Cardoso et al., 2007), which supports our results showing the performance of the coated seeds with a mixture of trehalose, *R. tropici*, and silk in high salinity levels.

### Physiological Response

Salt stress presents one of the most limiting abiotic factors that affect plant growth and production by altering physiological and biochemical activities (Rockwood et al., 2011; Hazzoumi et al., 2017). Protection of the photosynthetic machinery significantly contributes to the ability of the plant to withstand stress and reactive oxygen species (ROS) produced upon exposure to saline stress. This study demonstrated that the coated and primed seeds maintained several physiological processes under saline treatments, including photosynthesis through stomatal conductance and chlorophyll content. Many studies highlighted the effect of salinity on photosynthetic activity due to the sensitivity of Photosystem II to NaCl as a non-stomatal regulation (Brugnoli and Lauteri, 1991; Taïbi et al., 2016). While there is stomatal regulation that has also been reported for several plant species, both halophytes and non-halophytes, which refers to stomatal conductance, correlated with photosynthetic capacity (Wong et al., 1979). In this study, and to attenuate the effect of salinity on photosynthetic activity, seeds were treated with trehalose and *R. tropici*. Both directly affect photosynthesis by allocating and metabolizing carbohydrates (Gazzarrini and Tsai, 2014) and contributing to a higher N2 fixation rate (Bethlenfalvay et al., 1978), respectively.

Previous studies have shown that supplementation of trehalose in saline medium protects *Catharanthus roseus* from the inhibitory effects of salt on growth and photosynthesis (Crowe et al., 1984; Gazzarrini and Tsai, 2014; Hazzoumi et al., 2017). Trehalose accumulation was correlated with higher soluble carbohydrate levels, and a high photosynthesis capacity under stress and non-stress conditions, supporting the increase observed in non-saline treatment. In this study, variation in stomatal conductance and chlorophyll content were strongly expressed in high salinity levels, where coated seeds performed better than the treated seeds and the control, showing that combining trehalose and *R. tropici* helps to maintain a high photosynthetic activity. These increments probably may be attributed to the protective effects of trehalose on photosynthetic systems (Abdallah et al., 2016). Besides, it has been discovered that trehalose plays an important role in plant-microorganism interactions through a higher number of nodules and nitrogenase activity, greater biomass, and an increase in grain yield in inoculated plants under normal or stress conditions (Iturriaga et al., 2009). Photosynthetic and chlorophyll parameters in our study indicated that trehalose helps the plant mitigate the adverse effects of salinity and increase *R. tropici* performance under saline stress.

To tolerate salt ions, the plant accumulates polyphenols which are involved in the defense mechanism. The role of TPCs in the stimulation of plant resistance was provided in many species and for different biotic and abiotic stress situations (Benhamou et al., 2000). Phenolic compounds are directly involved in scavenging free radicals and protect plants against the damaging effects of increased ROS levels (Petridis et al., 2012). The negative impact of salinity was mainly attributed to water deficit due to lowered water potential in the root zone, specific ion toxicity arising from a higher concentration of sodium and chloride, and the nutritional imbalance.

The osmotic adjustment is a central part of the physiological mechanisms by which plants respond to salinity stress. The salinity generates water stress by disturbance of plant water balance (caused by ions disequilibrium). The accumulation of specific osmotic adjustment solutes (e.g., proline, soluble sugars, and soluble protein) in the cytosol and organelles helps in the osmotic adjustment and improving the growth and development of plants. We note that salt stress causes an increase in TPC accumulation. Increasing polyphenols content in tissue is reported to be a plant response to salinity and indicates the induction of synthesis of secondary metabolism to defend against salt stress. This considerable accumulation is confirmed in *Anethum graveolens* L. (Mehr et al., 2012) and *Vetiveria zizanioides* L. (Manebr et al., 2011). However, the plants treated with trehalose presents better performance in stressful environments. According to Sadak et al. (2019), trehalose foliar application in different concentrations (0,1 mM and 0,5 mM) improves the antioxidant defense system of the quinoa plant against reactive oxygen species (Sadak et al., 2019). The impact of trehalose was accompanied by a significant decrease in lipid peroxidation, hydrogen peroxide contents, and LOX (Lipoxygenase) activity. These decreases were correlated with significant increases in total phenolic contents as compared with untreated plants. A similar positive impact of trehalose was recorded in maize plants exposed to salt stress by increasing the growth parameters and photosynthetic activity of the plant (Rohman et al., 2019).

The antioxidant activity of phenolic compounds was principally due to their role as electron and hydrogen donors, which stabilize the unpaired electron (Huang et al., 2005). The stimulation of TPC by trehalose might be due to the role of this latter as signaling function and their implication in different metabolic pathways in stress conditions by inducing the synthesis of TPC (Alam et al., 2014; Sadak et al., 2019).

### Shoots and Root Development Under Salinity Stress

The present study includes the effect of seed treatments on photosynthetic activity through the regulation of stomata. Previous studies demonstrate that the effect of salinity on the CO_2_ assimilation rate was mainly due to the reduction of stomatal conductance (Brugnoli and Lauteri, 1991). Evidence of the impact on biomass production.

Exogenous trehalose application was effective in mitigating the harmful effects of saline stress conditions on gas exchange attributes. The ameliorative effect of trehalose on photosynthesis indicates that it plays a role in protecting the photosynthetic system, as already reported, through trehalose signaling mechanisms that can enhance this capacity. Furthermore, trehalose induces an ameliorative effect in plant biomass when carbon supply is not a limiting factor (Rady et al., 2011).

Such effects of exogenously applied trehalose might have been due to its contribution to lowering the cell osmotic potential, which helps plants absorb water from the soil. Plant length is one of the biomass components in addition to the total leaf area and dry biomass. This study demonstrated that seed treatments, under saline and normal conditions, help to maintain and improve plant biomass relative to the control. Other studies emphasized the role of trehalose in the regulation of Na^+^ in plant tissues, including leaves (Garcia et al., 1997), indicating that it may play a direct or indirect role in determining ion selectivity by controlling cellular exclusion of Na^+^. A hypothesis was supported by Joshi et al. (2020), where transgenic lines had higher K^+^/Na^+^ ratios than the wild type under salinity stress.

Our studies showed that coated and primed seeds and seedlings were less affected by saline stress in comparison to the control, as evidenced by their higher germination rates and better subsequent growth under these stress conditions due to efficient sequestration of sodium that has been considered as one of the critical components differentiating between control and treated seeds. Moreover, the degree of damage increases with stress intensity.

Based on the present results, it could be concluded that salinity stress adversely affected growth and physiological parameters compared with control plants. Trehalose can neutralize the effect of salinity stress and result in a significant improvement of morphological traits, physiological parameters, and phytochemical activity (Ding et al., 2018). Trehalose pretreatment alleviated damages through efficient stimulation of the antioxidant defense. Exogenous application of trehalose enhanced the level of internal trehalose, which mainly stimulated the protective activities for the plants (Abdallah et al., 2016).

The *P. vulgaris* root system undergoes significant architectural changes in root density and lateral root length due to the exogenous seed treatments. Those modifications in the root architecture may determine the capacity of the plant to acquire available water and nutrient. Analysis of plants from treated seeds showed that trehalose and *R. tropici* increase the root density. It also promotes the principal and lateral root formation under saline treatments. The same result was observed for coated seeds, indicating the effect of trehalose and the bacteria after 3 months of conservation at 4°C. Lateral root development has been suggested to be under the control of polar auxin transport (Casimiro et al., 2001). Many studies have confirmed that trehalose is an essential signal metabolite in plants, linking growth and development to carbon metabolism (Figueroa and Lunn, 2016). A recent study suggests that auxin acts downstream of trehalose 6 Phosphate to promote seed filling, thereby providing an outstanding example of how a metabolic signal governs the hormonal control in a critical phase transition in a crop plant (Meitzel et al., 2021).

## Conclusion

Exogenous application of trehalose and *R. tropici*, ingredients of the seed coating, impacts plant growth under non-stressed and stressed environments. The roots readily absorb trehalose and are easily transported to the aerial parts to function as a major defensive response to several abiotic stresses. This experiment is evidence of the role of trehalose in seed priming. This sugar induces osmotic stress that stimulates the growth of shoots and roots. This study proves that fresh soaking seems to be more productive. Using silk-based coating can be more efficient for preserving the formulation in stored seeds and delivering it to seedlings. This study shines a new light on the role of trehalose in the biofertilizers preservation, plant survival under osmotic stress conditions, and the development of new functional seed coatings that integrate Rhizobium preservation with precise delivery in the soil.

## Data Availability Statement

The raw data supporting the conclusions of this article will be made available by the authors, without undue reservation.

## Author Contributions

BM and ATZ contributed to the coating design and formulation at MIT. LK managed the project at the UM6P. YZ contributed by funding and scientific follow-up from OCP. MM and ZH contributed in the physiological and agronomic evaluation of the seed coating at the UM6P. All authors contributed to the article and approved the submitted version.

## Conflict of Interest

YZ was employed by the company OCP Group. The remaining authors declare that the research was conducted in the absence of any commercial or financial relationships that could be construed as a potential conflict of interest.

## Publisher's Note

All claims expressed in this article are solely those of the authors and do not necessarily represent those of their affiliated organizations, or those of the publisher, the editors and the reviewers. Any product that may be evaluated in this article, or claim that may be made by its manufacturer, is not guaranteed or endorsed by the publisher.
