# Supplementary figures and images for: Bioformulation of Silk-Based Coating to Preserve and Deliver Rhizobium tropici to Phaseolus vulgaris Under Saline Environments
Source: Front Plant Sci. 2021 Aug 2;12:700273. doi: 10.3389/fpls.2021.700273 (PMC8366584; doi:10.3389/fpls.2021.700273)

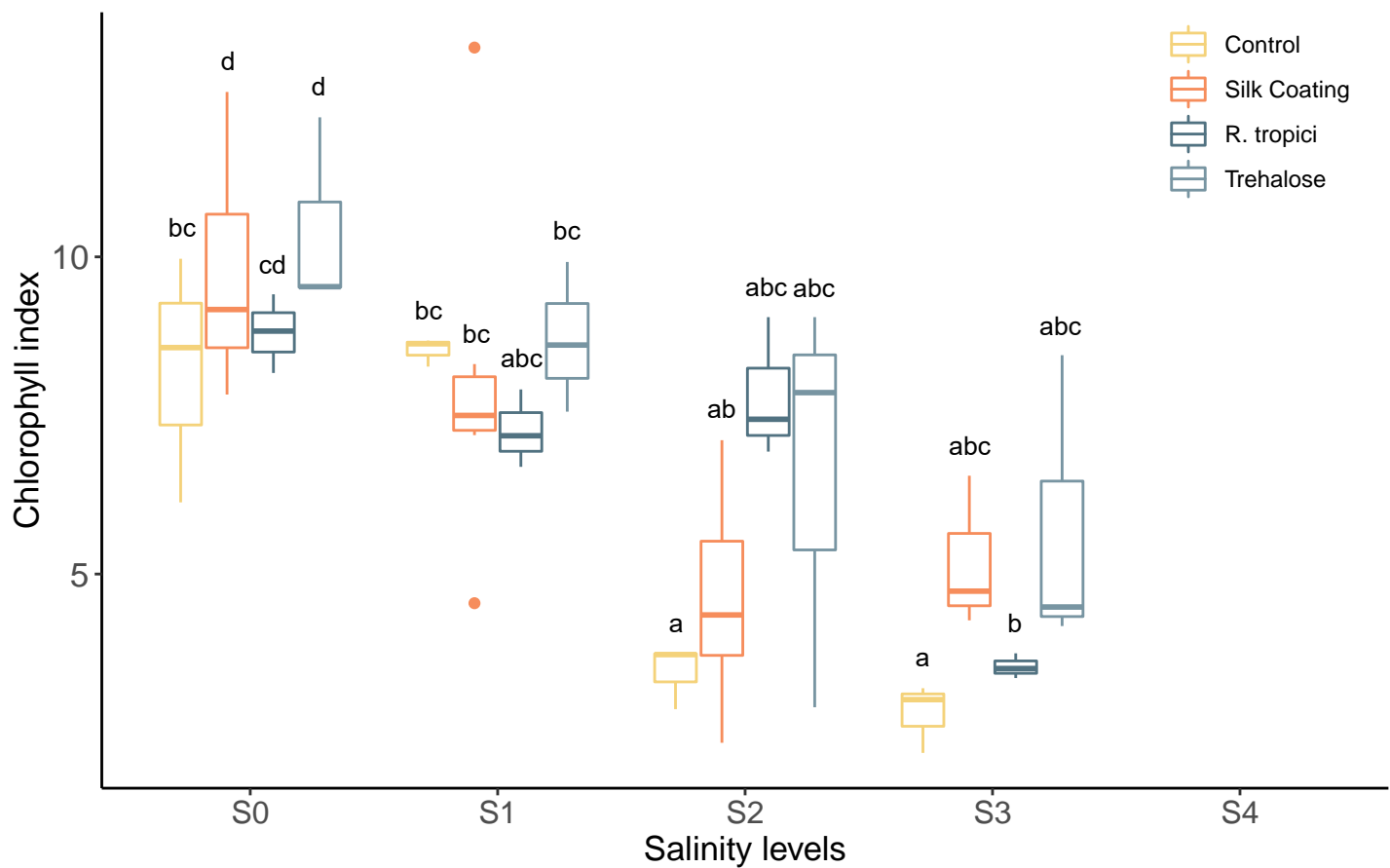

Supplement: Supplementary file 1 [file Data_Sheet_1.PDF]

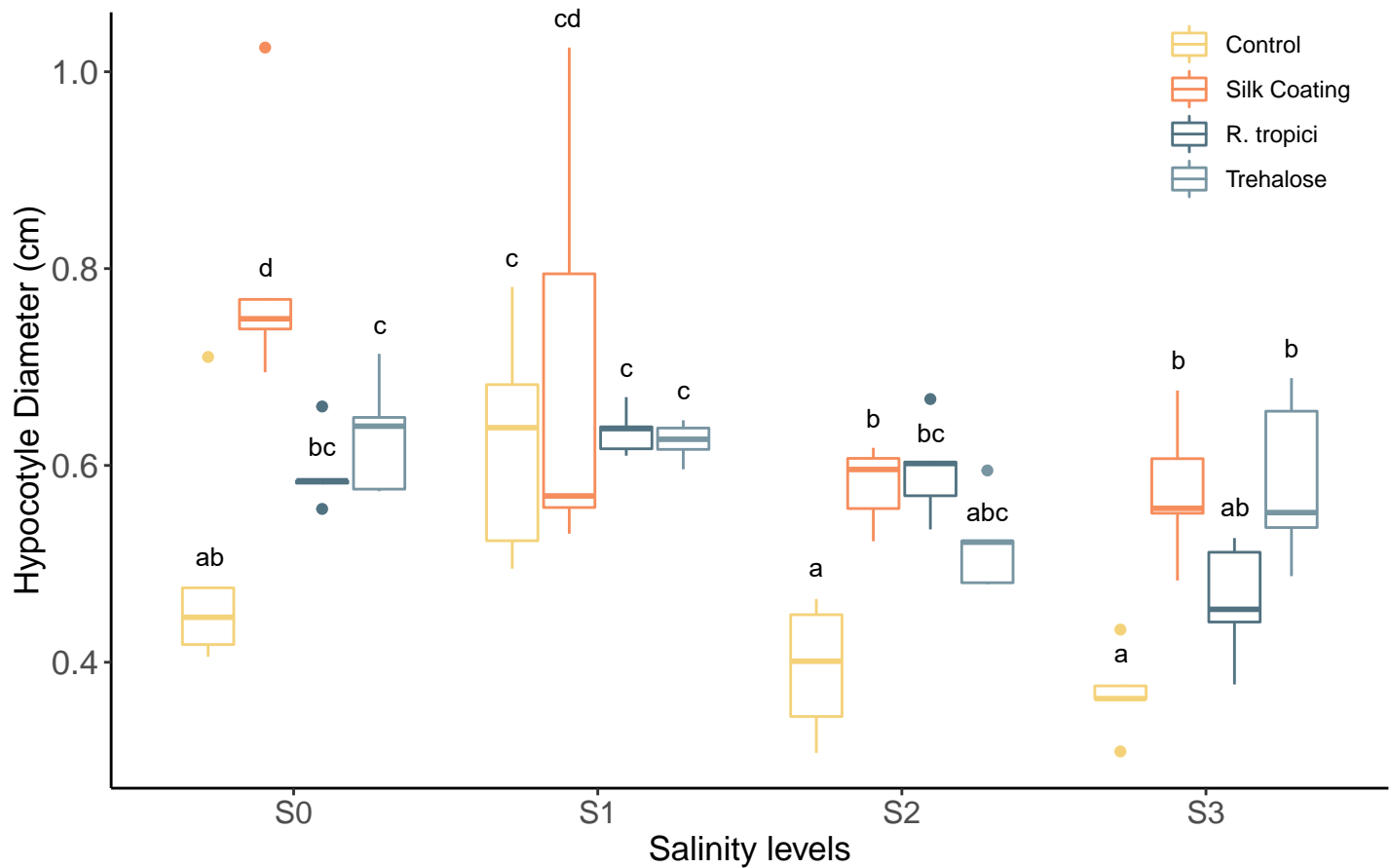

Supplement: Supplementary file 2 [file Data_Sheet_2.PDF]

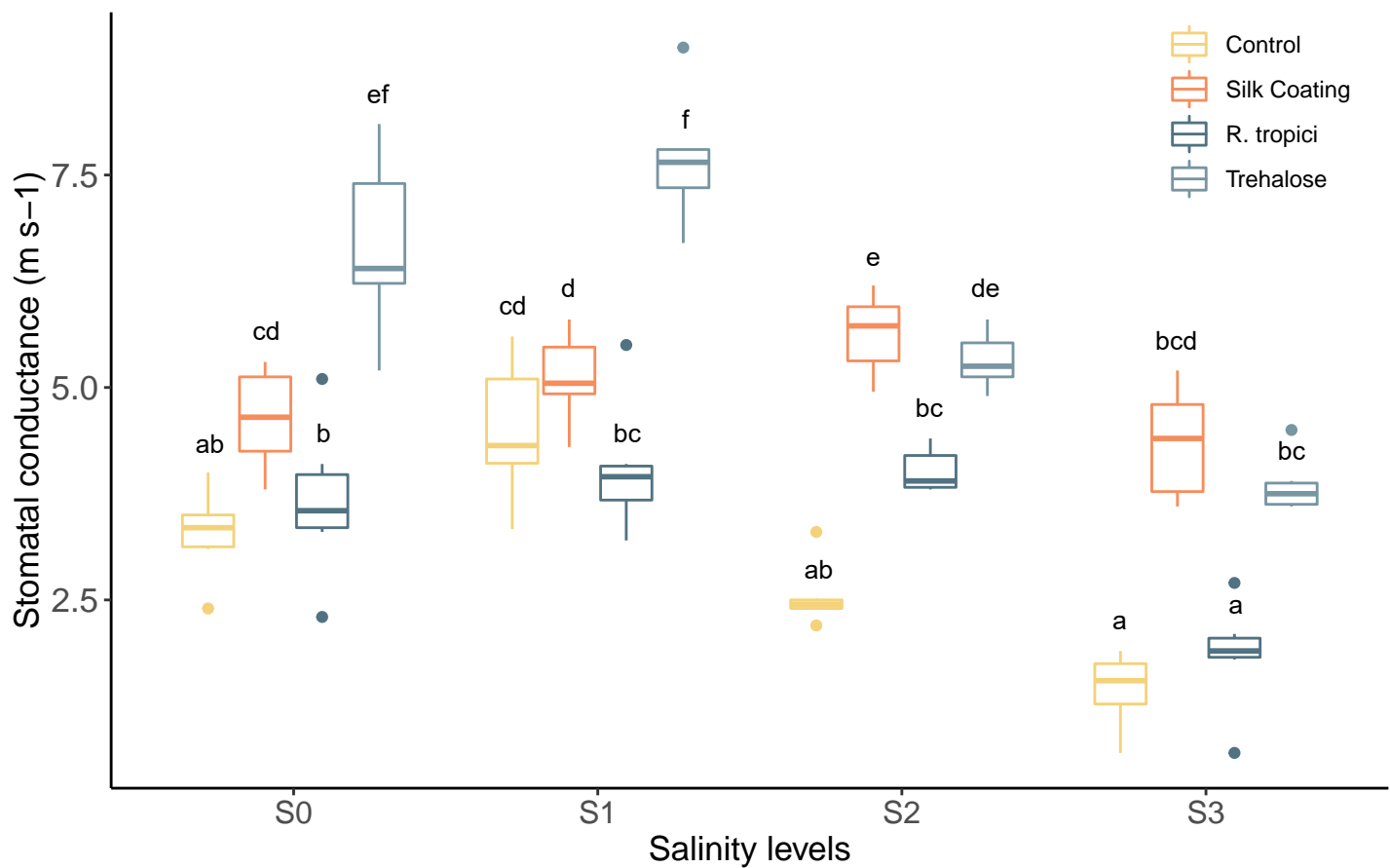

Supplement: Supplementary file 3 [file Data_Sheet_3.PDF]

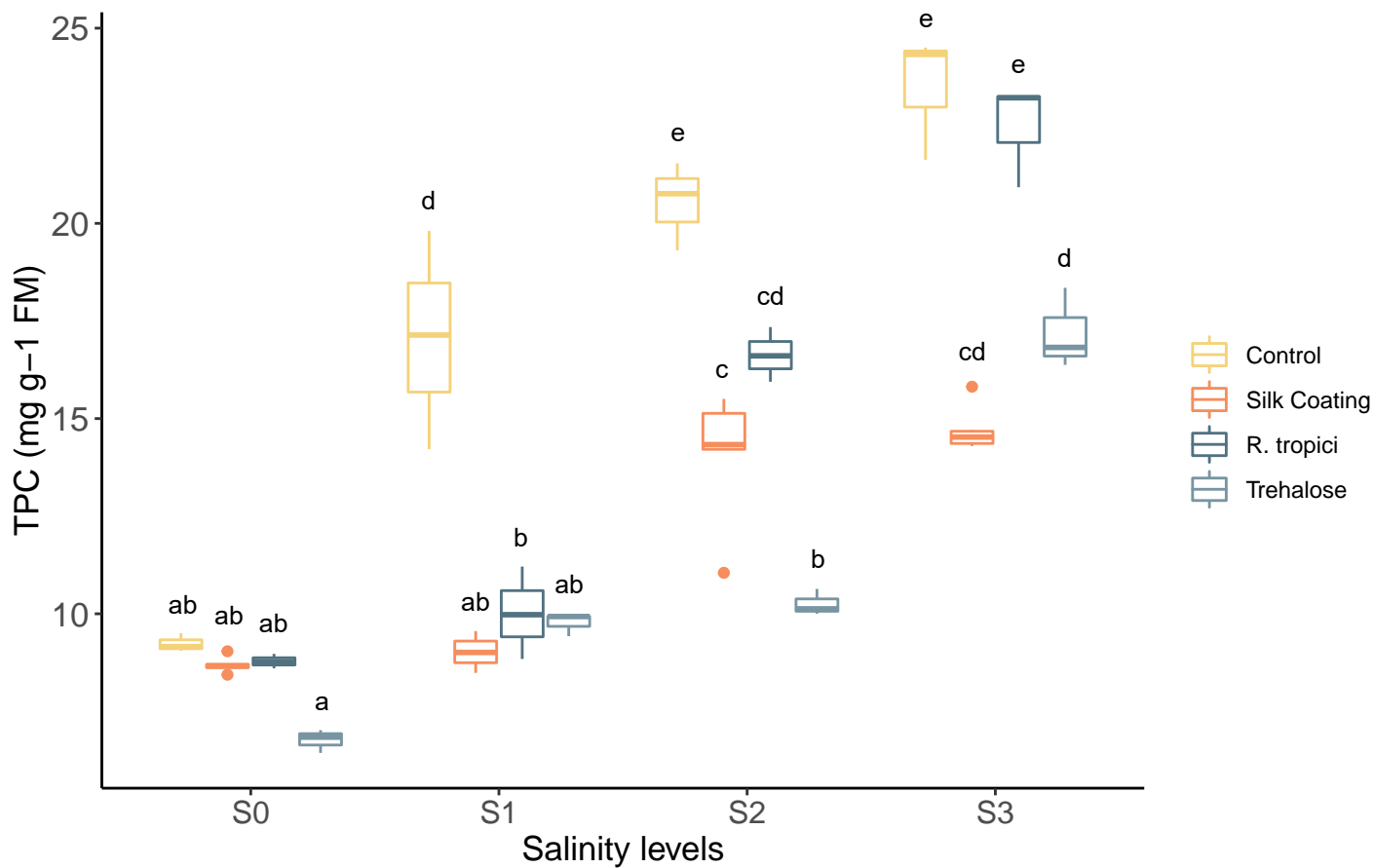

Supplement: Supplementary file 4 [file Data_Sheet_4.PDF]

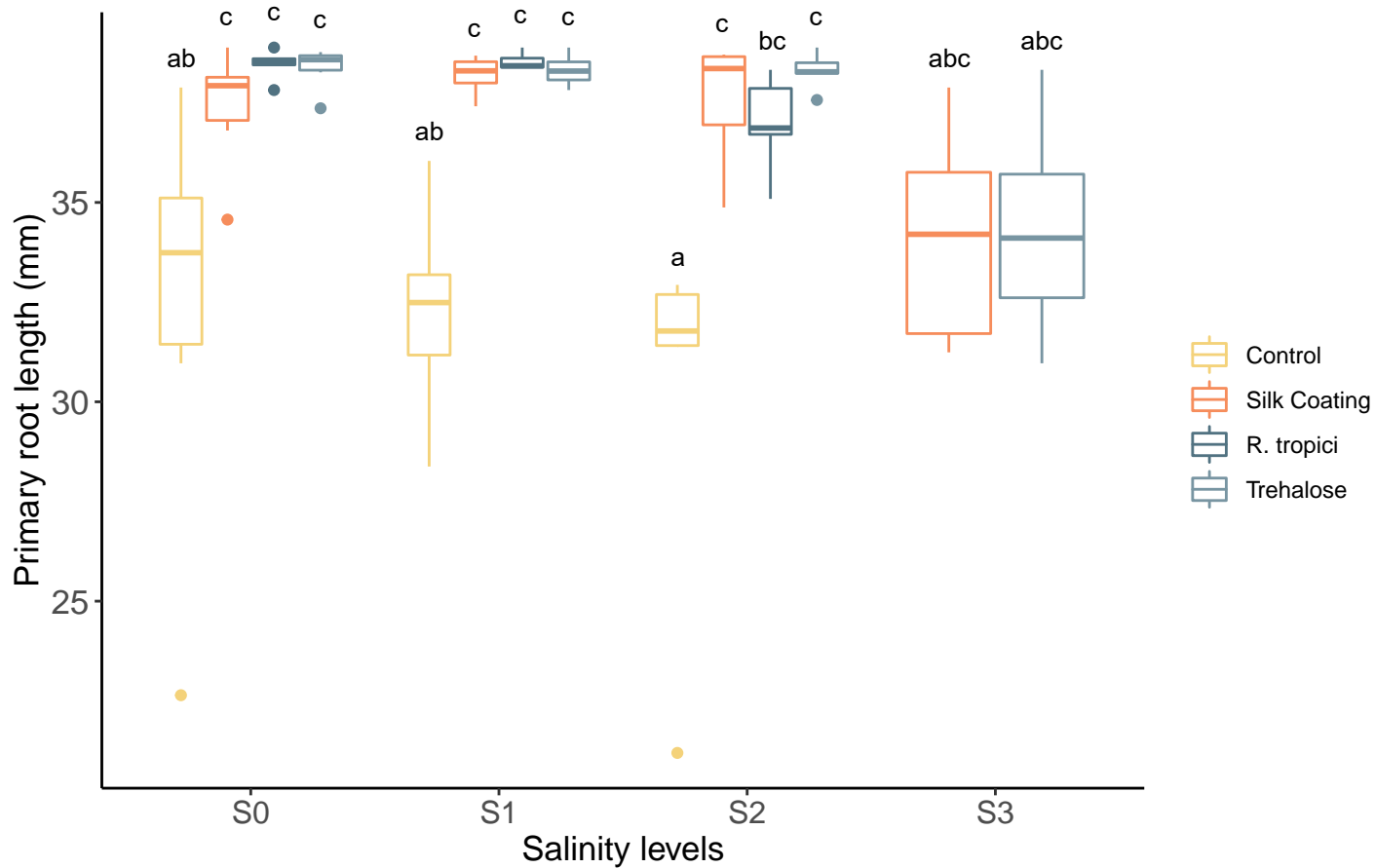

Supplement: Supplementary file 5 [file Data_Sheet_5.PDF]

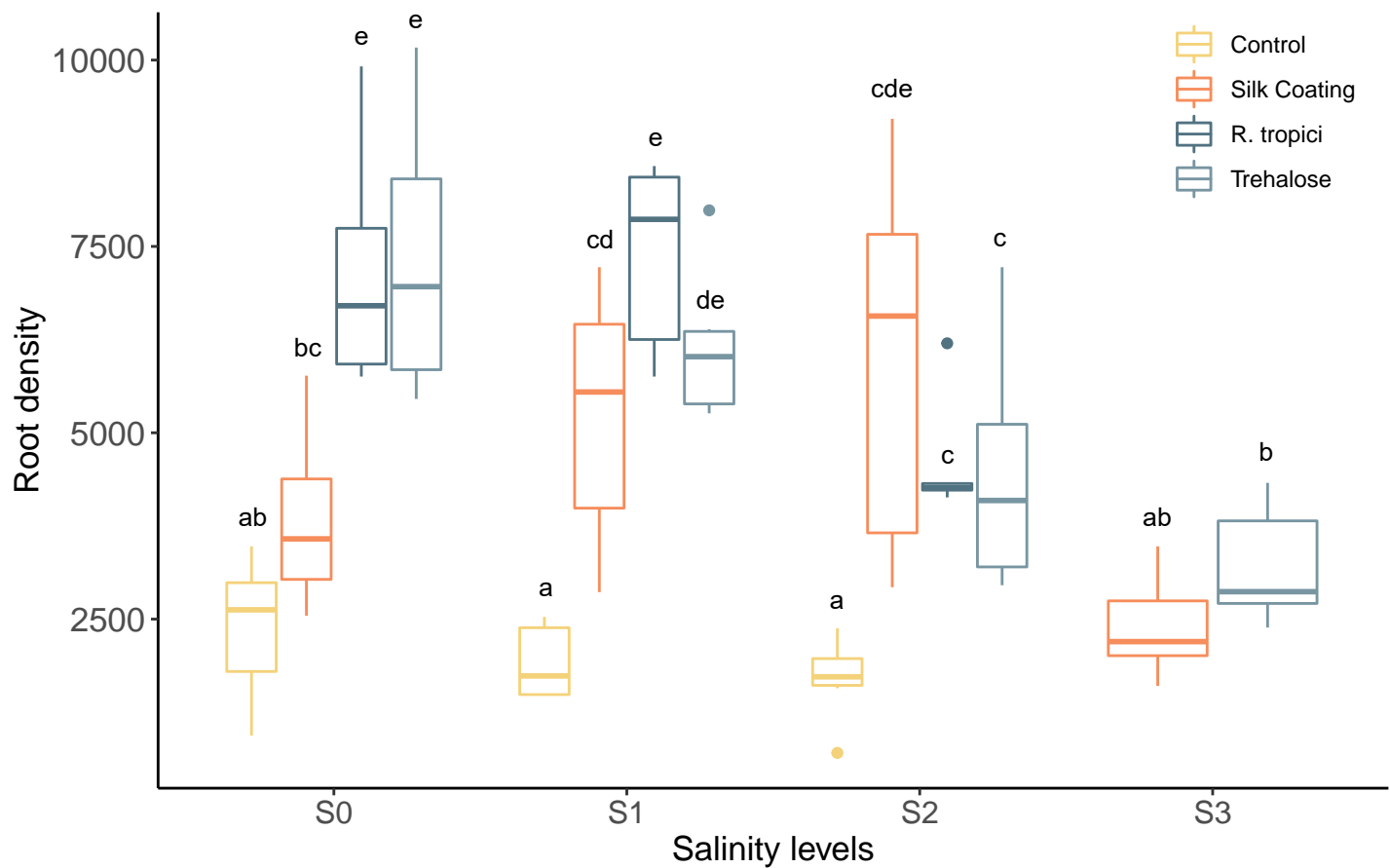

Supplement: Supplementary file 6 [file Data_Sheet_6.PDF]

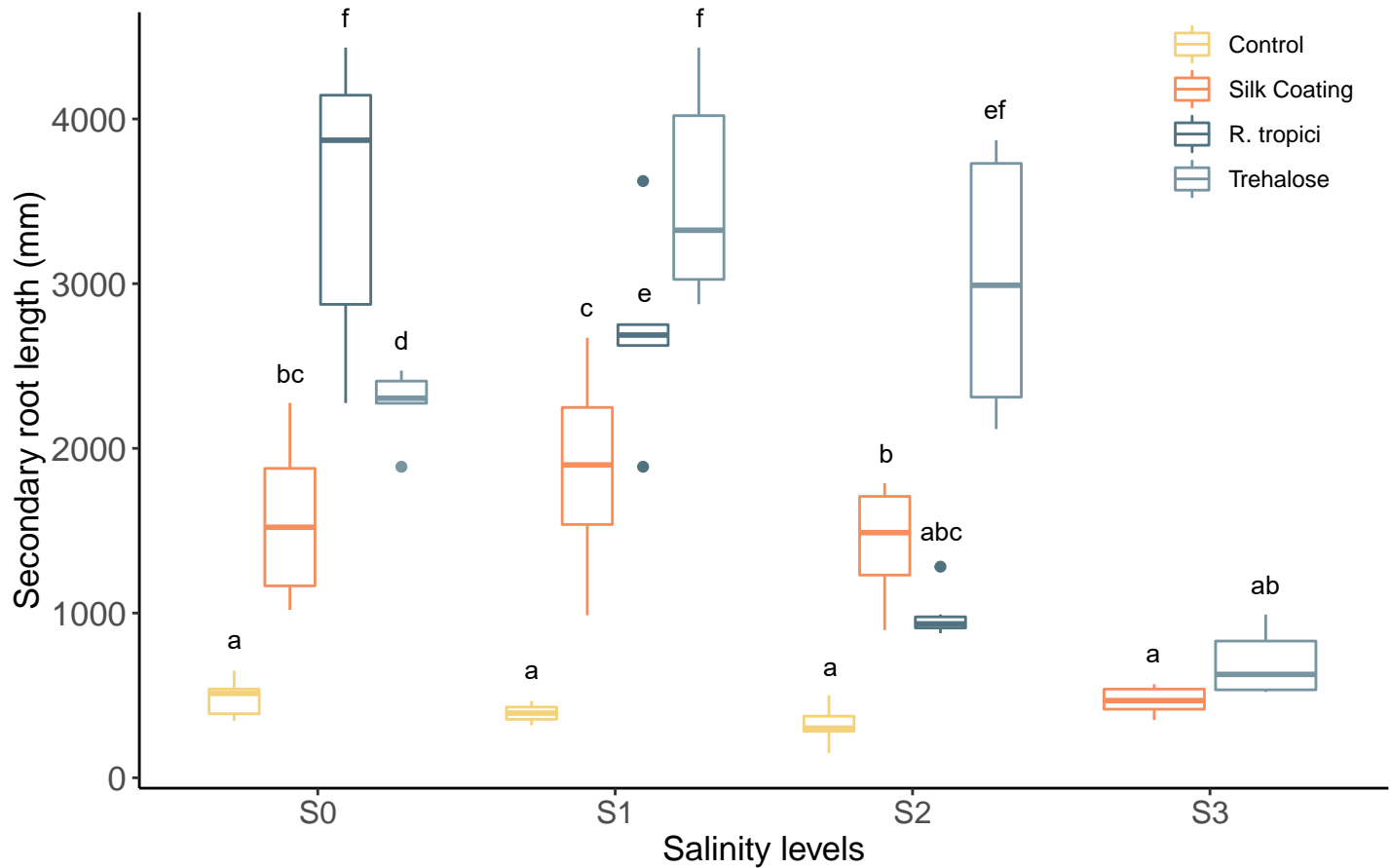

Supplement: Supplementary file 7 [file Data_Sheet_7.PDF]

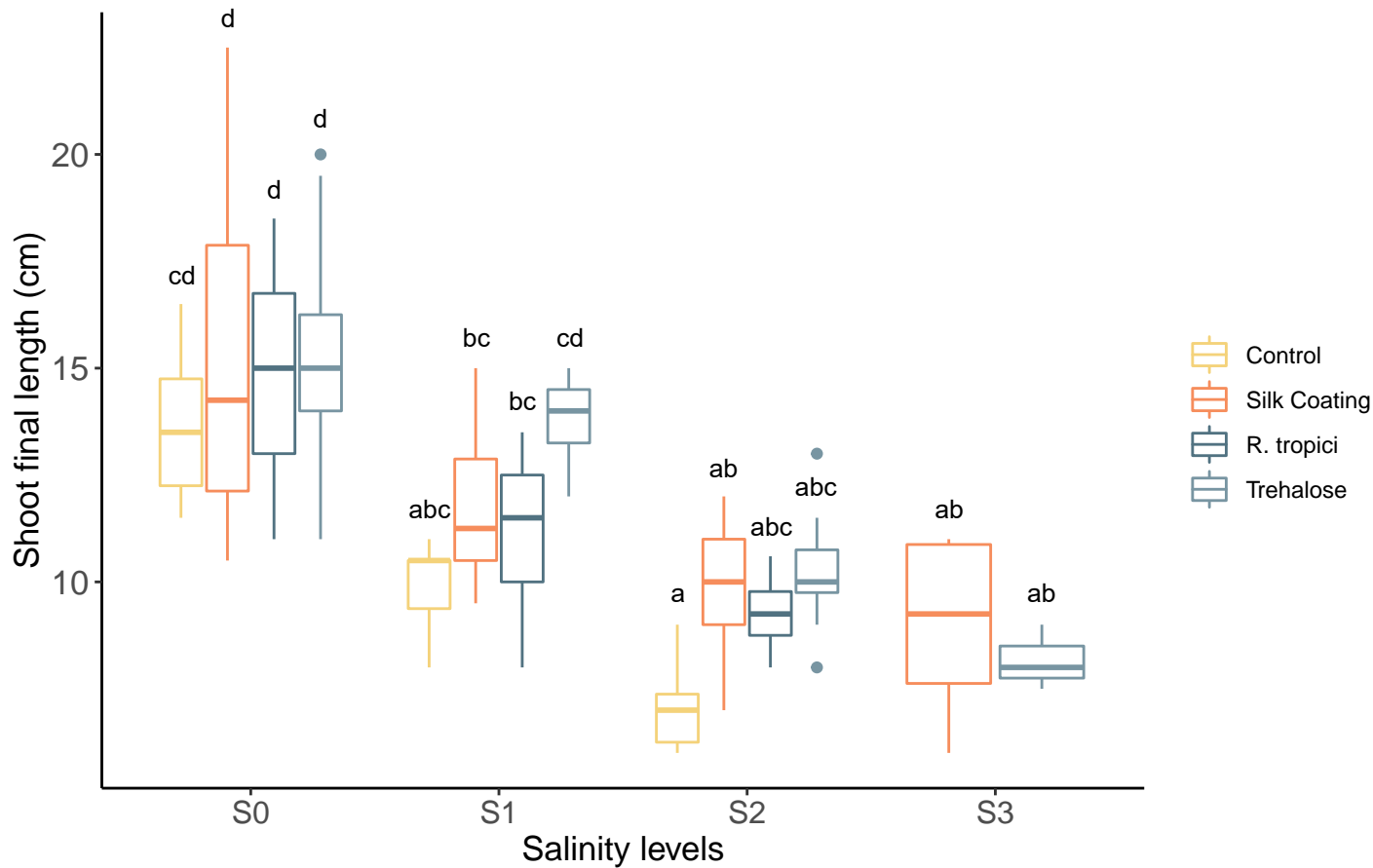

Supplement: Supplementary file 8 [file Data_Sheet_8.PDF]
